# Supplementary material for: Regulatory T Cell Depletion Using a CRISPR Fc-Optimized CD25 Antibody
Source: Int J Mol Sci. 2022 Aug 5;23(15):8707. doi: 10.3390/ijms23158707 (PMC9369266; doi:10.3390/ijms23158707)
Supplement: Supplementary file 1 [file ijms-23-08707-s001.zip › ijms-1808960-supplementary.pdf]

Rattus norvegicus: Rn Celera

Chr.6 (AC_0000741)

139,140,597 – 139,140,001

TGGGCTTCACTGTTGTCCAAACTGTGTGCAGATTATGGCCCATGGGTAAGAGGTTTAGCATTAGAACACAGATACCCACATTGGACAATGGT

GGGGGAACACAGATACCCATACTGCAAGGCTCTTCGGGCCCTTTCCTAAAAGTGTACTAGGAGTGGGACTGGGCTCAAAGGGATTAGGTGTG

ATCTGGCCTGGTGAGGCTGACACTGACAAGCCCAATGGTTGGGTGTTGCATCCTCCATTTATACAGCC**AGG**GACTTGGGGAGGGTACAAAAT

GGAGGACTTGTAGGAGCTTGGGTCCAGACCTGTCAGACAAAATGATCACGCATACTTTTTCTTGTAGCTGAAACAACAGCCCCATCTGTCTA

TCCACTGGCTCCTGGAACTGCTCTCAAAAGTAACTCCATGGTGACCCTGGGATGCCTGGTCAAGGGCTATTTCCCTGAGCCAGTCACCGTGA

CCTGGAACTCTGGAGCCCTGTCCAGCGGTGTGCACACCTTCCCAGCTGTCCTGCAGTCTGGGCTCTACACTCTCACCAGCTCAGTGACTGTA

CCCTCCAGCACCTGGCCCAGCCAGACCGTCACCTGCAACGTAGCCCACCCGGCCAGCAGCACCAAGGTGGACAAGAAAATTGGTGAGAGAAC

AACCAGGGGACGAGGGGCTCACTAGAGGTGAGGATAAGGCATTAGACTGCCTACACCAACCAGGGTGGGCAGACATCACCAGGGAGGGGGCC

TCAGCCCGGGAGACCAAACATTCTCCTTTGTCTCCCTTCTGGAGATTTCTATGTCCTTTACACCCATTTATTAATATTCTGGGTAAGATGCC

CTTGCATCATGACATACAGAGGCAGACTAGAGTATCAACCTGCAAAAGGTCATACCCAGGAACAACCTGCCATGATCCCACACCAGAACCAA

CCTGGTGCCTTCTAACCTATAGACACCAATAACACACAGCCTTCTCTCTGCAGTGCCCAGAAACTGTGGAGGTGATTGCAAGCCTTGTATAT

GTACAGGTAAGTTACTAGGCCTTAAATTCCAGCCCCAGGTCCAACAAATGTCCTCTGAGGCCCATGTTGGAGGATATTGGCATATTTCCACC

TTTCTTCTTCATCTACAGGCTCAGAAGTATCATCTGTCTTCATCTTCCCCCCAAAGCCCAAAGATGTGCTCACCATCACTCTGACTCCTAAG

Supplementary Figure S1. Genomic sequence of rat IgG1 heavy chain locus. Genomic sequence of rat IgG1 heavy chain immunoglobulin locus with the exons for the CH1, Hinge, CH2 and CH3 highlighted in grey. The PAM sequence for gRNA-R1 (AGG) is underlined and highlighted in yellow.

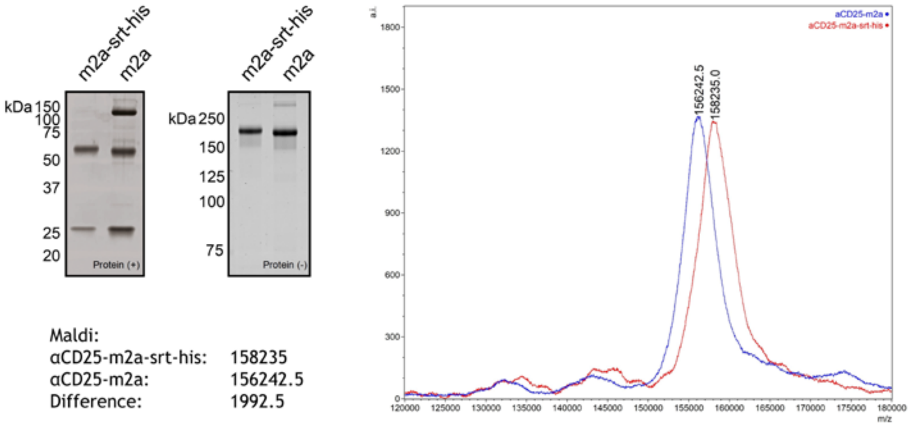

Supplementary Figure S2. Sortase mediated his-tag removal leads to covalent inter heavy chain linkage in αCD25-m2a. Reducing SDS-PAGE (left gel) of αCD25-m2a suggests generation of side product from sortase-mediated reaction (Fig 1E). Non-reducing SDS-page (right gel) and mass spectrometry measurements indicate monomeric antibody product, suggesting sortase mediated reaction results in covalent bond forming between heavy chains of single molecules.

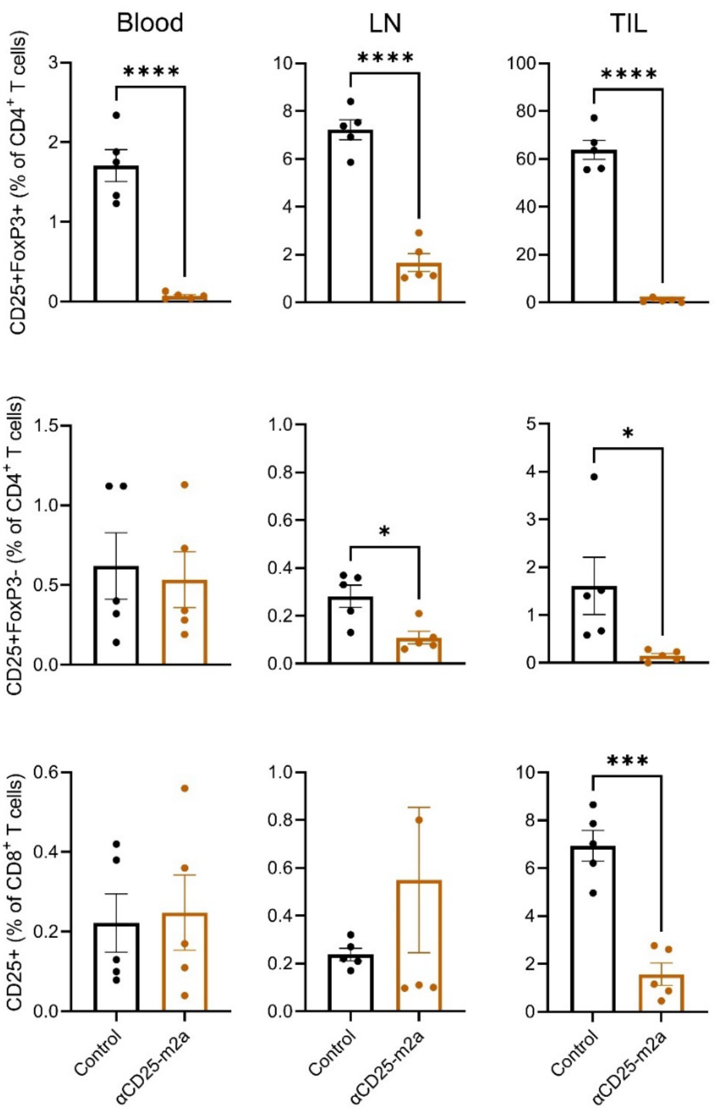

Supplementary Figure S3. CD25⁺ T-cell subsets in blood, lymph nodes and tumor from mice treated with αCD25-m2a or an isotype control. Figure is supplementary to figure 2C. The percentage $CD25^+FOXP3^+$ of $CD4^+$ T-cells, percentage $CD25^+FOXP3^-$ of $CD4^+$ T cells and the percentage of $CD25^+$ of $CD8^+$ T-cells are shown. Data is represented as the mean ± SEM. Statistical significance was determined using an unpaired two-tailed Student's T test and is shown as * = p<0.05, ** = p<0.01, and *** = p<0.001.

Supplementary Table S1: HDR Donor construct

Element	Sequence
5' HA	AGGGCTGCGTATGAGCCACAAGTGAATCCTGACCCAAGAATAGAGAGTGCTAAACCTACTTACATCAAAGCCAACTGAAAGGACAAGGCCAGCAAAACGAAGCTAAGGCCAGAGATCTTGGACTATGAAGAGTTCAGAGAACCTAGGATCAGGAACCATTAGTGAACA<br>GACAAAGGCAGGTAAAGCAGCCTAGGAGTGGACAAAGACAGGAGAATACAGAAGACGGCAGGGATGACCCGACTTCAGTTTGGGCTTCACTGTTGTCCAAACTGTGTGCAGATTATGGCCCATGGGTAAGAGGTTTAGCATTAGAACACAGATACCCACATTGGACAAT<br>GGTGGGGGAACACAGATACCCATACTGCAAGGCTCTTCGGGCCCTTTCCTAAAAGTGTACTAGGAGTGGGACTGGGCTCAAAGGGATTAGGTGTGATCTGGCCTGGTGAGGCTGACACTGACAAGCCCAATGGTTGGGTGTTGCATCCTCCATTTATACAGCC
mIgG2a CH1-CH3	GCTAGCGATCGCAGGCGCAATCTTCGCATTTCTTTTTTCCAGCTAAGACTACCGCTCCTAGCGTTTATCCTTTGGCCCCTGTATGTGGGGACACCACTGGCTCTTCTGTAACTCTGGGCTGTTTGGTGAAAGGGTACTTTCCAGAACCTGTAACTTTGACATGGAATTCAGG<br>GTCTCTCTCTTCAGGAGTACATACTTTTCCCGCAGTTTTGCAAAGCGATCTTTACACACTTAGTTCCTCCGTCACCGTTACTTCTTCTACCTGGCCCAGTCAGTCCATAACATGTAACGTGGCCCATCCTGCCAGCAGCACAAAAGTAGACAAAAAAATCGAACCTAGAGG<br>CCCTACTATTAAGCCCTGCCCACCATGCAAATGCCCAGCCCCAAATCTCCTCGGTGGTCCTAGCGTCTTCATCTTCCCCCCCAAGATTAAGGATGTGCTGATGATTTCATTGAGCCCAATTGTCACATGTGTGGTCGTGGATGTGTCAGAGGATGACCCTGACGTGCAAAT<br>ATCTTGGTTTGTAAATAACGTAGAGGTGCATACCGCTCAGACTCAGACTCACCGGGAGGACTATAATAGCACTCTCAGGGTGGTCTCCGCACTTCCAATTCAGCACCAGGACTGGATGTCCGGCAAAGAGTTCAAGTGTAAAGTCAATAACAAGGATTTGCCCGCACCA<br>ATAGAACGGACCATCTCTAAACCTAAAGGGAGTGTACGCGCCCCACAGGTTTACGTGCTGCCCCCACCCGAGGAGGAAATGACCAAAAAGCAGGTGACACTCACCTGCATGGTTACCGATTTTATGCCCGAAGACATATATGTTGAGTGGACTAACAACGGGAAGACC<br>GAGCTGAATTATAAAAATACCGAACCCGTTTTGGACTCAGATGGCTCATACTTCATGTACTCCAAACTCCGGGTAGAGAAAAAGAACTGGGTTGAAAGAAACAGCTACTCATGCAGCGTGGTGCATGAGGGGCTCCACAATCATCATACCACCAAGTCTTTCTCACGGA<br>CACCTGGGAAACGTACGGGAA
G4S,LPETGG,H6	CGTACGGGAGGCGGAGGCAGCCTGCCGGAAACCGGCGGCCATCATCATCATCATCATTGA
IRES	GTCGACGTCGAGGCCCCTCTCCCTCCCCCCCCCCTAACGTTACTGGCCGAAGCCGCTTGGAATAAGGCCGGTGTGCGTTTGTCTATATGTTATTTTCCACCATATTGCCGTCTTTTGGCAATGTGAGGGCCCGGAAACCTGGCCCTGTCTTCTTGACGAGCATTCCTAGGG<br>GTCTTTCCCCTCTCGCCAAAGGAATGCAAGGTCTGTTGAATGTCGTGAAGGAAGCAGTTCCTCTGGAAGCTTCTTGAAGACAAACAACGTCTGTAGCGACCCTTTGCAGGCAGCGGAACCCCCCACCTGGCGACAGGTGCCTCTGCGGCCAAAAGCCACGTGTATAAGA<br>TACACCTGCAAAGGCGGCACAACCCCAGTGCCACGTTGTGAGTTGGATAGTTGTGGAAAGAGTCAAATGGCTCTCCTCAAGCGTATTCAACAAGGGGCTGAAGGATGCCCAGAAGGTACCCCATTGTATGGGATCTGATCTGGGGCCTCGGTGCACATGCTTTACATGTG<br>TTTAGTCGAGGTTAAAAAAACGTCTAGGCCCCCCGAACCACGGGGACGTGGTTTTCCTTTGAAAAACACGATGATAATATGGCCACA
Bsr	GCCACCATGGCCAAGCCTTTGTCTCAAGAAGAATCCACCCTCATTGAAAGAGCAACGGCTACAATCAACAGCATCCCCATCTCTGAAGACTACAGCGTCGCCAGCGCAGCTCTCTCTAGCGACGGCCGCATCTTCACTGGTGTCAATGTATATCATTTTACTGGGGGAC<br>CTTGTGCAGAACTCGTGGTGCTGGGCACTGCTGCTGCTGCGGCAGCTGGCAACCTGACTTGTATCGTCGCGATCGGAAATGAGAACAGGGGCATCTTGAGCCCCTGCGGACGGTGCCGACAGGTGCTTCTCGATCTGCATCCTGGGATCAAAGCCATAGTGAAGGACAG<br>TGATGGACAGCCGACGGCAGTTGGGATTCGTGAATTGCTGCCCTCTGGTTATGTGTGGGAGGGCTAAGT
SV40 polyA	ACTAGTCGAGTGTGCCTTCTAGTTGCCAGCCATCTGTTGTTTGCCCCTCCCCCGTGCCTTCCTTGACCCTGGAAGGTGCCACTCCCACTGTCCTTTCCTAATAAAATGAGGAAATTGCATCGCATTGTCTGAGTAGGTGTCATTCTATTCTGGGGGGTGGGGTGGGGCAGGA<br>CAGCAAGGGGGAGGATTGGGAAGACAATAGCAGGCATGCTGGGGATGCGGTGGGCTCTATGGA GATCTTGTACA
3'HA	CAAAATGGAGGACTTGTAGGAGCTTGGGTCCAGACCTGTCAGACAAAATGATCACGCATACTTTTTCTTGTACCTGAACAACAGCCCCATCTGTCTATCCACTGGCTCCTGGAACTGCTCTCAAAAGTAACTCCATGGTGACCCTGGGATGCCTGGTCAAGGGCTATTTC<br>CCTGAGCCAGTCACCGTGACCTGGAACTCTGGAGCCCTGTCCAGCGGTGTGCACACCTTCCCAGCTGTCCTGCAGTCTGGGCTCTACACTCTCACCAGCTCAGTGACTGTACCCTCCAGCACCTGGCCCAGCCAGACCGTCACCTGCAACGTAGCCCACCCGGCCAGC<br>AGCACCAAGGTGGACAAGAAAATTGGTGAGAGAACAACCAGGGGACGAGGGGCTCACTAGAGGTGAGGATAAGGCATTAGACTGCCTACACCAACCAGGGTGGGCAGACATCACCAGGGAGGGGGCCTCAGCCCGGGAGACCAAACATTCTCCTTTGTCTCCCTT
